# A Biomimetic Elastic SEI Film Enables Micron‐Sized Bismuth Anodes for Wide‐Temperature Sodium‐Ion Batteries

**DOI:** 10.1002/advs.78740

**Published:** 2026-11-09

**Authors:** Zhenzhen Dang, Jing Li, Jiayue Gao, Qiuyu Sun, Ning Pan, Yangyang Li, Jingfeng Ning, Hongqing Yin, Jiaye Ye, Weijia Meng

**Affiliations:** ^1^ School of Smart City and New Energy Xi'an Jiaotong University City College Xi'an China; ^2^ Shaanxi Provincial Key Laboratory of New Transportation Energy and Automotive Energy Saving School of Energy and Electrical Engineering Chang'an University Xi'an China; ^3^ China Energy Engineering Group Hydrogen Energy Co. Ltd Beijing China; ^4^ School of Chemistry and Physics Faculty of Science Queensland University of Technology Brisbane Queensland Australia

**Keywords:** alloy, anode, bismuth, chemical engineering, materials science, modulus, nanocrystal, particle, polymerization, solvation

## Abstract

Micron‐sized bismuth (µ‐Bi) is a promising high‐capacity alloy anode for sodium‐ion batteries, but its ∼250% volume expansion causes particle pulverization and continuous SEI destruction, especially at low temperatures. Inspired by the alternating hard inorganic/soft organic layers of seashells, this work in situ constructs a biomimetic elastic SEI on µ‐Bi via microscopic solvation engineering. The G2/NaBF_4_ electrolyte is designed to promote the formation of a flexible polyether‐like organic component and reinforcing NaF‐containing inorganic domains through complementary interfacial decomposition pathways, thereby constructing a mechanically adaptive organic–inorganic SEI. The biomimetic SEI has a low Young's modulus of 334 MPa and a broad elastic transition, efficiently dissipating volumetric stress. Consequently, the µ‐Bi anode delivers 100% capacity retention after 450 cycles at 5C and 25°C, stable cycling at 9.49 mg cm^−2^, and excellent wide‐temperature rate/cycling performance at −40°C. This biomimetic interfacial design offers a new strategy to overcome volume‐expansion failure in micron‐sized alloy anodes.

## Graphical abstract

Inspired by natural seashells' hard‐soft layered structure, a biomimetic elastic SEI is in situ constructed on micron‐sized Bi via a G2/NaBF_4_ electrolyte. The flexible polyether‐based organic layer and rigid NaF nanocrystals synergistically dissipate volumetric stress, enabling a low Young's modulus. This µ‐Bi anode delivers 100% capacity retention after 450 cycles at 5C and excellent −40°C performance.

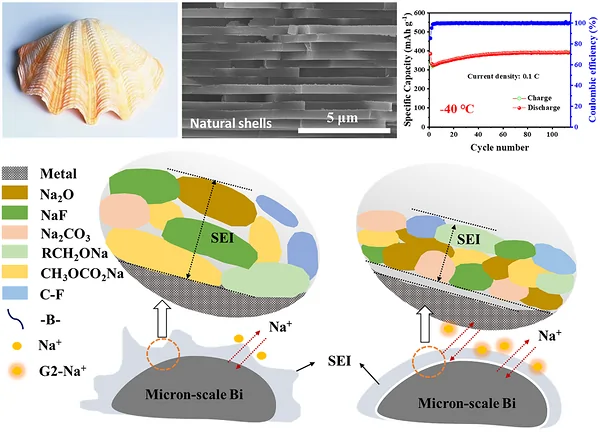

## 1 Introduction

With the global transformation of the energy structure, the efficient integration of large‐scale renewable energies (such as wind and solar energy) and the rapid development of smart grids and distributed energy storage systems have imposed urgent demands on energy storage devices with high energy density, low cost, and high safety [1, 2, 3, 4, 5, 6]. Among various candidate systems, sodium‐ion batteries (SIBs) are widely recognized as a highly promising large‐scale energy storage technology due to the abundant reserves, uniform geographical distribution, and low cost of sodium resources in the Earth's crust, as well as their intercalation/deintercalation chemistry similar to that of mature lithium‐ion batteries [3, 7, 8, 9, 10]. However, the hard carbon anodes widely studied suffer from a relatively low theoretical specific capacity (typically around 300 mAh g^−1^), making it difficult to meet the rigorous requirements of next‐generation high‐energy‐density battery systems [9, 11, 12, 13].

To break through the capacity bottleneck, alloy‐type anode materials with high theoretical specific capacities (such as metallic bismuth Bi, with a high theoretical specific capacity of 385 mAh g^−1^) have attracted widespread attention from academia and industry [14, 15]. Among them, micron‐sized bismuth powder (µ‐Bi), compared to nano‐sized materials, features a higher tap density, simpler synthesis processes, lower specific surface area, and excellent potential for industrial‐scale applications [16, 17]. Nevertheless, during repeated sodiation and desodiation electrochemical cycles, µ‐Bi undergoes severe volume expansion and contraction of up to ∼250% [18]. Such tremendous mechanical stress induces severe stress concentration within the micron‐sized particles, subsequently triggering rapid particle pulverization and collapse of the electrode structure [19, 20]. More fatally, in conventional electrolytes, the unstable solid electrolyte interphase (SEI) film on the surface of the micron‐sized particles undergoes a vicious cycle of repeated cracking and regeneration under drastic deformation [21]. This dynamic cracking process continuously consumes active sodium ions and the electrolyte, resulting in low Coulombic efficiency, sharp impedance escalation, and ultimately catastrophic capacity decay of the battery [22].

Particularly in extremely low‐temperature environments (such as −40°C), the solvation structure in conventional electrolytes tends to rigidify, leading to sluggish desolvation kinetics; simultaneously, conventional SEI films become highly embrittled at low temperatures, with ion transport barriers increasing exponentially [8, 23, 24]. This poses dual severe challenges of macroscopic volumetric stress and microscopic interfacial kinetics for µ‐Bi anodes operating in wide‐temperature regimes, especially under ultra‐low‐temperature conditions. Current strategies in academia addressing the volume expansion of alloy anodes primarily focus on nanotechnology engineering and carbon‐based composite matrix construction. However, nanotechnology engineering not only significantly increases the preparation cost and process complexity of materials, but its large specific surface area also aggravates side reactions and reduces the initial Coulombic efficiency [25, 26]. Therefore, how to directly start from electrolyte system design without complex nano‐modification, and in situ construct a biomimetic SEI film with both high mechanical strength (to resist pulverization) and strong elastic buffering capacity (to absorb stress) on the surface of micron‐sized active particles via microscopic solvation structure regulation, has become a key scientific issue to break through the application bottleneck of micron‐sized alloy anodes.

Although electrolyte solvation engineering has been widely investigated to regulate Na^+^ coordination, desolvation kinetics, and interfacial chemistry, the relationship between electrolyte decomposition pathways and the mechanical adaptability of the resulting SEI remains an important consideration for micron‐sized alloy anodes. In particular, the interphase must accommodate substantial volume changes while maintaining structural integrity and continuous Na^+^ transport. These requirements highlight the need to coordinate interfacial chemistry with mechanical design, rather than optimizing ionic transport or interphase rigidity alone. An important question is whether the solvent and salt can be selected to generate complementary organic and inorganic components in situ, thereby integrating deformation accommodation and structural reinforcement within a single interphase. Conventional ester‐based electrolytes (such as 1 M NaClO_4_ in ethylene EC/DEC, designated as the NED electrolyte) decompose to form conventional SEI films primarily composed of rigid inorganic carbonates (such as Na_2_CO_3_) and inorganic dimers [27, 28]. Such conventional interfacial films lack sufficient elasticity and ductility, making them susceptible to macroscopic brittle cracking under the severe volumetric stress of micron‐sized particles. Inspired by the unique brick‐and‐mortar microstructures of natural seashells (such as abalone shells and nacre), whose unique alternating layered structure of a hard inorganic layer (providing macroscopic compressive hardness) and a soft organic layer (providing microscopic elastic buffering and energy dissipation) can effectively impede microcrack propagation [29, 30].

Herein, we propose a microscopic solvation engineering strategy for the in situ construction of a biomimetic elastic SEI film. We designed an electrolyte system (designated as the BG electrolyte) using diethylene glycol dimethyl ether (DEGDME, abbreviated as G2) as the solvent and sodium tetrafluoroborate (NaBF_4_) as the sodium salt. Thorough theoretical calculations and multi‐dimensional experimental characterizations confirm that G2 solvent molecules undergo radical polymerization triggered by specific weak‐bond homolysis at low potentials, generating a flexible polyether‐based organic sodium salt matrix to construct the soft organic layer of the biomimetic interface. Meanwhile, the defluorination reduction of NaBF_4_ salt delivers high‐modulus inorganic NaF nanocrystals to the interface to construct the hard inorganic scaffold of the biomimetic interface, suggesting the rigid‐flexible coupled microarchitecture of seashells. Ex situ PeakForce QNM atomic force microscopy (AFM) and nanoindentation measurements confirm that the Young's modulus of this biomimetic SEI film is only 334 MPa, which is far lower than the 1691 MPa of conventional ester‐based SEI films, and exhibits a broad non‐linear elastic deformation transition region capable of efficiently absorbing and dissipating the volumetric stress of µ‐Bi particles. Consequently, the µ‐Bi anode based on this biomimetic SEI film exhibits exceptional comprehensive electrochemical performance, retaining a high capacity retention of 100% after 450 ultra‐high‐rate cycles at 5C at 25°C, stable long‐term cycling under a high mass loading (9.49 mg cm^−2^), and excellent wide‐temperature cycling stability and rate responses under extreme ultra‐low temperatures of −40°C. This study not only resolves the interfacial failure problem caused by volume expansion in micron‐sized alloy anodes, but also provides a novel biomimetic perspective for the interfacial chemical design of high‐energy‐density and wide‐temperature sodium‐ion batteries in the future.

## 2 Results and Discussion

### 2.1 Design Concept and Theoretical Calculation Analysis

Natural shells achieve extremely high fracture toughness through a hierarchical, layered, alternating structure of inorganic hard layers and organic soft layers (as shown in Figure 1a–c). Our proposed microscopic solvation engineering is based precisely on this structural philosophy. By selecting DEGDME as the solvent and NaBF_4_ as the solute in the electrolyte, we induced the formation of a rigid‐flexible interfacial layer on the µ‐Bi surface (compare with the conventional ester‐based SEI in Figure 1d and the bioinspired SEI in Figure 1e). To systematically demonstrate the feasibility of the formation mechanism of the biomimetic elastic SEI film from thermodynamic and molecular dynamics scales, density functional theory (DFT) calculations were utilized to thoroughly evaluate the frontier molecular orbital energy levels, spatial charge distribution, and bond dissociation energies (BDE) of solvents and salt molecules in different electrolyte systems [31], along with an in‐depth theoretical deduction of their reduction decomposition pathways (relevant data and reaction pathways are detailed in Figures S1–S3 and Equations S1–S20). In the NaBF_4_/DEGDME system, BDE calculation results indicate that specific chemical bonds such as C1‐O1 and C2‐O1 in the G2 solvent molecule possess lower homolytic cleavage energies (only 3.72 and 3.76 eV, respectively; Figure S1). When the battery is charged to the low‐potential region of the anode, G2 molecules enriched at the interface and gaining electrons readily undergo homolytic cleavage along these low‐energy‐barrier weak bonds, subsequently triggering in situ polymerization via continuous radical coupling reactions (Equations S1–S7) [32, 33]. This polymerization process directionally generates a substantial amount of flexible polyether‐based organic sodium salt matrices (chemical formula: NaO‐[CH_2_CH_2_O]_n_‐Na), forming the soft organic buffer layer within the biomimetic interface. Concurrently, NaBF_4_ salt undergoes thermodynamic dissociation (NaMF_n_ ⇌ NaF + MF_n‐1_ where M = B or P, Equation S1) and subsequent electrochemical reduction reactions, continuously supplying high‐shear‐modulus and mechanically robust inorganic NaF nanocrystals to the interface (Equation S16) to serve as the hard inorganic scaffold of the biomimetic interface [34, 35, 36].

**FIGURE 1:**
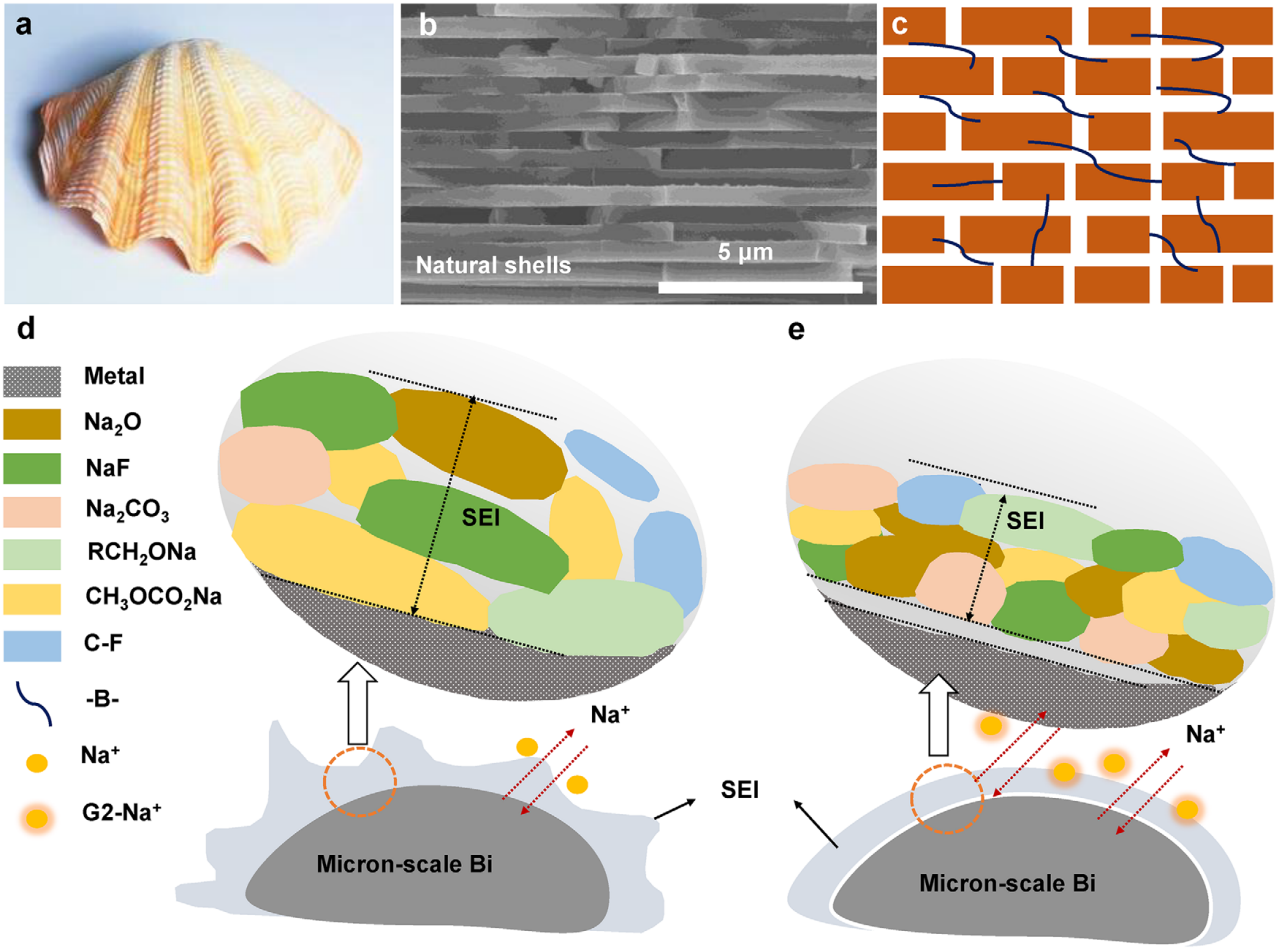
Schematic illustration of the biomimetic elastic SEI design for µ‐Bi anodes in wide‐temperature SIBs. (a) Digital photograph of a natural seashell, (b) cross‐sectional SEM image showing the unique hard inorganic layer/soft organic buffer layer alternating hierarchical layered structure, and (c) its corresponding microstructural scheme. (d) Schematic diagram of the fragile conventional SEI formed on a µ‐Bi anode in a conventional commercial ester‐based electrolyte; (e) Schematic diagram of the robust biomimetic elastic SEI formed on a µ‐Bi anode in the optimized BG electrolyte.

In contrast, in the commercial system of NED, EC can also undergo ring‐opening reactions via the low‐bond‐energy O2‐C2 bond cleavage (Figure S2), but according to its subsequent reduction decomposition pathways (Equations S8–S10), its primary reduction products are rigid alkyl sodium carbonate dimers [(CH_2_OCO_2_Na)_2_] and massive Na_2_CO_3_ inorganic particles [33, 34, 35, 36]. These products are overly rigid yet lack flexibility and sufficient ductility, rendering the resulting conventional SEI film extremely brittle and susceptible to macroscopic brittle cracking when µ‐Bi particles undergo drastic anisotropic deformation [37, 38]. Moreover, inevitable trace moisture and other impurities in the electrolyte participate in the interfacial evolution process. As shown in Equations S11–S13, the stepwise reduction of trace water and CO_2_ contributes to partial Na_2_O and Na_2_CO_3_ inorganic phases, while the hydrolysis byproduct HF further promotes the uniform in situ precipitation of high‐purity NaF nanocrystals (Equations S17–S20) [39]. The stable fluctuation curves of total system energy and temperature vs. time derived from ab initio molecular dynamics (AIMD) simulations (Figure S4) further verify the high reliability of the simulated system under thermodynamic equilibrium states. The aforementioned theoretical calculations and multi‐step reaction pathway derivations support that utilizing the BG system can precisely mimic the microstructural construction philosophy of natural seashells, in situ cultivating a biomimetic interfacial layer composed of a flexible polyether organic network and high‐mechanical‐strength/high‐ion‐conductivity inorganic nanophases on the surface of micron‐sized bismuth anodes, laying a solid theoretical foundation for exhibiting exceptional wide‐temperature stability in subsequent electrochemical tests.

DEGDME was selected as the model ether solvent because its multiple ether oxygen atoms provide coordination sites for Na^+^, while its flexible molecular backbone and calculated C─O bond dissociation energies provide a plausible basis for the formation of G2‐derived interfacial species under reductive conditions. In combination with NaBF_4_, the proposed decomposition pathways can generate organic species that accommodate deformation and inorganic fluoride‐containing phases that contribute structural reinforcement. The low glass‐transition temperature of the BG electrolyte (−86.9°C) further supports its suitability for low‐temperature operation. The comparative tests of different sodium salts in DEGDME also help identify the NaBF_4_‐based formulation as the most favorable among the formulations examined in this study. However, because a systematic comparison of different ether solvents was not performed, the present results establish the suitability of the G2/NaBF_4_ combination rather than its universal superiority over other ether‐based electrolytes.

### 2.2 Solvation Structure and Physicochemical Properties Characterization

To thoroughly reveal how the microscopic solvation structure of the electrolyte dictates macroscopic electrochemical performance, systematic AIMD simulations were first performed on both electrolyte systems. Snapshot configurations presented in Figure 2a,b clearly illustrate the distinct local coordination microenvironments of Na^+^ in the NED and BG systems. Radial distribution function g(r) analyses (Figure 2c,d) demonstrate that in the BG system, Na^+^ forms a more stable inner solvation sheath with ether oxygen atoms in G2 molecules and BF_4_^−^ anions. Coordination number statistics (Figure 2e) further confirm that this system significantly optimizes the solvation structure of Na^+^ through specific anion/solvent cooperative coordination modes; such a tight and stable structure effectively promotes the uniform and dense formation of the interfacial film during the initial charging cycle [40].

**FIGURE 2:**
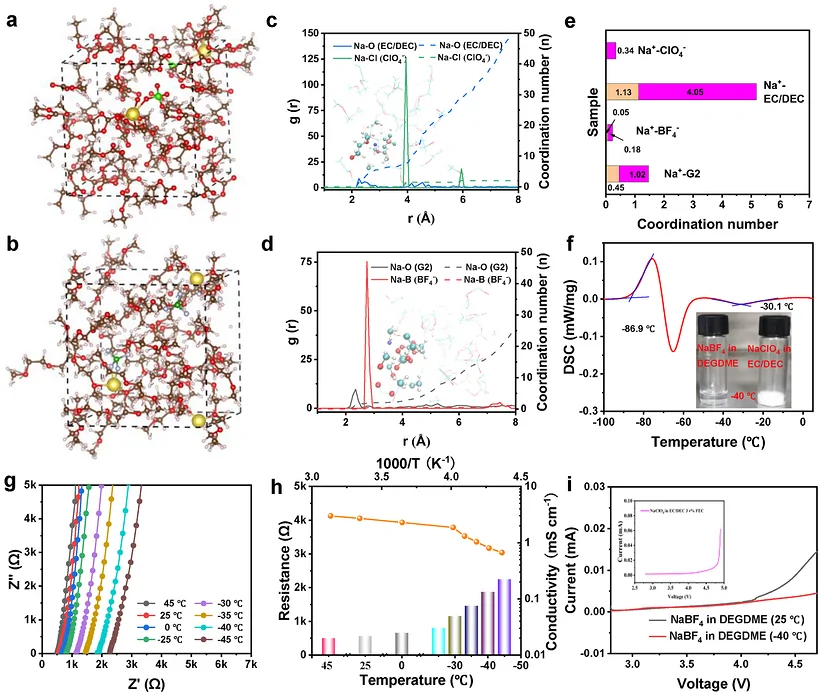
Solvation structure simulation and wide‐temperature physicochemical characterization of electrolytes. (a,b) Snapshots of Na^+^ solvation structures in NED and BG systems derived from AIMD simulations; (c,d) Corresponding radial distribution functions g(r) and coordination number analyses of Na^+^; (e) Composition statistics of the primary solvation shell of Na^+^ under different electrolyte environments; (f) DSC thermal analysis curves of both electrolytes (insets show their physical states at −40°C); (g) EIS curves of the NaBF_4_/DEGDME electrolyte measured from 45°C to −45°C; (h) Arrhenius plots of ionic conductivity as a function of temperature; (i) LSV curves at different temperatures evaluating the electrochemical stability windows.

Superior ultra‐low‐temperature operational capability is one of the core advantages of this electrolyte system. Differential scanning calorimetry (DSC) results indicate that the glass transition temperature of the BG electrolyte is extremely low at −86.9°C, far superior to that of the commercial NED electrolyte (−30.1°C; Figure 2f). Even under extreme freezing conditions of −40°C, the BG electrolyte maintains a favorable liquid state, thereby completely circumventing the macroscopic physical blockage of ion conduction pathways caused by low‐temperature crystallization. To evaluate charge transport kinetics, alternating current (AC) impedance responses of the electrolytes were measured over a wide temperature range from 45°C to −45°C (Figure 2g). Arrhenius fitting of the impedance data (Figure 2h) and subsequent variable‐temperature electrochemical impedance spectroscopy analyses confirmed that the electrolyte maintains a low ion transport activation energy and high bulk ionic conductivity even at cryogenic temperatures. Linear sweep voltammetry (LSV) measurements (Figure 2i) indicate that at extremely low temperatures of −40°C, the BG system still possesses a wide electrochemical stability window (>4.2 V vs. Na/Na^+^) without noticeable electrolyte decomposition side reactions. Such wide‐temperature physicochemical stability endowed by microscopic solvation regulation provides an indispensable premium electrochemical environment for constructing a biomimetic elastic SEI film on the electrode surface [41].

### 2.3 Systematic Electrochemical Performance of µ‐Bi Anode

To comprehensively evaluate the enhancement efficacy of the biomimetic elastic SEI film on electrode electrochemical kinetics and cycling stability, systematic electrochemical tests were conducted on the µ‐Bi electrode (original SEM morphology and XRD phase characterization of its commercial powder are shown in Figure S5). Cyclic voltammetry (CV) curves reveal multiple sharp, symmetric, and highly reversible redox peaks for µ‐Bi within the voltage range of 0.01–1.5 V (Figure 3a), demonstrating exceptional reversibility of the alloying/dealloying reactions of sodium ions within the micron‐sized bismuth crystal lattice. Compared with the erratic charge–discharge behavior in commercial electrolytes (Figure S6), Figure 3b further confirms the remarkably stable voltage plateau characteristics in the BG system, with the charge–discharge curves for the first five cycles overlapping closely, reflecting the outstanding structural self‐adaptation and stability achieved by the biomimetic SEI film immediately after formation in the first cycle.

**FIGURE 3:**
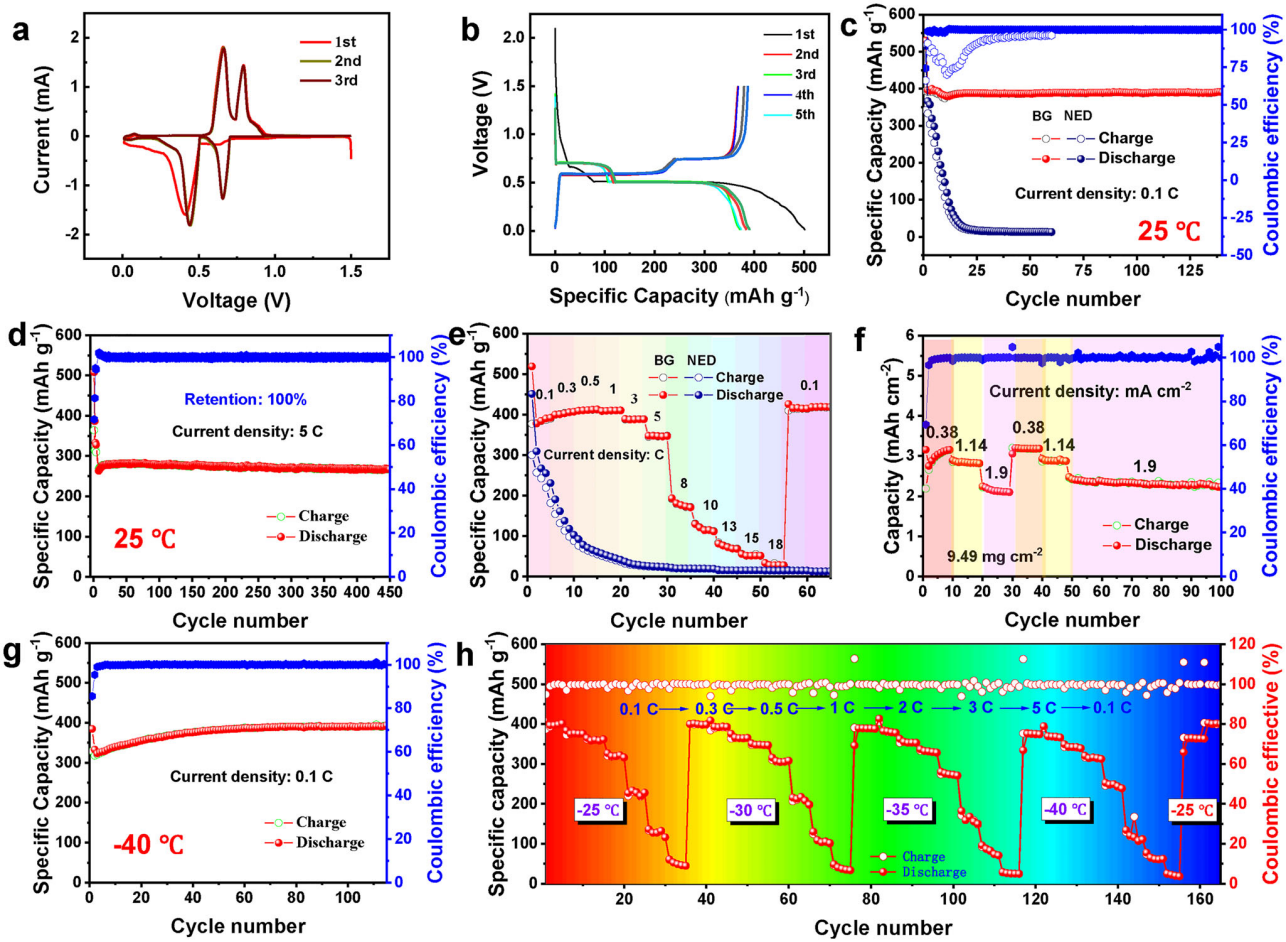
Electrochemical performance characterization of the µ‐Bi anode in the BG electrolyte. (a) CV curves revealing excellent redox reversibility; (b) Galvanostatic charge–discharge voltage profiles at various cycles; (c) Long‐term cycling performance at 0.1 C at 25°C; (d) Long‐term cycling stability and 100% capacity retention at a high rate of 5 C at 25°C; (e) Rate performance evaluation at stepwise current densities; (f) Cycling performance under a high active mass loading; (g) Cycling stability at an extreme ultra‐low temperature of −40°C; (h) Rate performance tests across a wide temperature range (from −25°C to −40°C).

Under ambient conditions at 25°C, the µ‐Bi electrode exhibits outstanding cycling lifespan. At a low rate of 0.1 C (1 C = 385 mA g^−1^), the cell delivers a high specific capacity close to the theoretical limit with virtually zero capacity decay over 130 consecutive cycles (Figure 3c). More impressively, even under a high current density of 5C for long‐term lifespan testing, the electrode achieves efficient cycling for up to 450 cycles with a capacity retention as high as 100% (Figure 3d). Simultaneously, the electrode exhibits excellent rate capability (Figure 3e and Figure S7), maintaining a capacity of 115 mAh g^−1^ at a high rate of 10C and rapidly recovering its capacity when the current density returns to 0.1C, verifying that micron‐sized particles undergo no pulverization or electrical contact loss under high‐rate impact. Furthermore, the electrode operates stably under harsh conditions of high active mass loading (9.49 mg cm^−2^, Figure 3f) and high areal capacity (3.2 mAh cm^−2^, Figure S8). Long‐term galvanostatic cycling tests of symmetrical cells (Figure S9) indicate that symmetrical cells utilizing the BG electrolyte possess superior polarization stability compared to the commercial control group, successfully suppressing dendrite growth and interfacial breakdown. Tested within a narrower voltage window (0.01–1 V), the µ‐Bi electrode maintains a discharge capacity of 380 mAh g^−1^ at 0.125C and completes 2000 ultra‐long cycles at 5C (Figure S10).

The breakthrough advantage of this biomimetic SEI film is most prominently manifested in extreme ultra‐low‐temperature environments. Under cryogenic conditions of −40°C, the µ‐Bi electrode preserves stable charge–discharge voltage plateaus (Figure 3g), maintaining stable capacity over a hundred cycles at 0.1C. In variable‐temperature rate capability tests (Figure 3h), the cell releases theoretical capacities corresponding to current density variations across a wide temperature gap from −25°C to −40°C, demonstrating strong low‐temperature adaptability. In addition, comparative evaluations of alternative common sodium salts in DEGDME solvents (such as NaPF_6_, NaOTF, and their dual‐salt composite systems with NaBF_4_) were tested as supplementary tests (Figures S11–S13). The results reveal that, whether under ambient high‐rate or low‐temperature environments, the optimized BG single‐salt system exhibits superior comprehensive electrochemical metrics. The initial Coulombic efficiencies (ICE) of µ‐Bi electrodes in different electrolytes were further evaluated to compare their first‐cycle electrochemical reversibility (Figure S14). The ICE values of 1 M NaBF_4_/DEGDME, 1 M NaPF_6_/DEGDME, 1 M NaOTf/DEGDME, and 0.9 M NaOTf + 0.1 M NaBF_4_/DEGDME are 73%, 60%, 63%, and 75%, respectively. Although the mixed‐salt electrolyte exhibits a slightly higher ICE than the BG electrolyte, the BG formulation delivers better overall electrochemical performance in the subsequent rate, cycling, and low‐temperature tests. These results indicate that the ICE alone is insufficient to assess the long‐term performance of alloy anodes, and that the stability and mechanical adaptability of the resulting interphase must also be considered. To clearly elucidate the crystallographic nature of multi‐step phase evolution in the bismuth anode during discharge–charge processes, ex situ XRD measurements were performed (Figure S15). By collecting phase patterns of electrode sheets at key selected potential nodes of charge–discharge, the ex situ XRD profiles intuitively display the reversible phase evolution kinetics of the initial hexagonal Bi phase transforming into a series of intermediate phases (such as Na_3_Bi alloy phases) during sodiation [42]. The results demonstrate high structural reversibility of the alloying reaction in the BG electrolyte, perfectly recovering the initial Bi phase upon charging without accumulating irreversible byproduct crystals, which strongly confirms from a crystallographic perspective that the biomimetic SEI film effectively protects the internal lattice integrity of the active material [43].

### 2.4 Chemical Composition and Microstructure of Biomimetic SEI

To thoroughly elucidate the formation mechanism of the biomimetic SEI film, the energetic properties of various electrolyte components were first calculated using DFT (Figure 4a,b). Frontier molecular orbital energy level analysis indicates that the energy level alignment of the BG system effectively induces selective reduction of solvents on the anode surface [44]. Calculated solvation energy barriers (Figure 4c) further verify that this system reduces the activation energy of reduction reactions, thereby facilitating the synergistic deposition of flexible organic components and highly conductive inorganic components. Combined with the reaction pathway deductions in Figures S1–S3 and Equations S1–S20, the SEI evolution process can be clearly outlined: (1) Formation of organic components (Soft Layer): The low cleavage energy of C─O bonds in the DEGDME solvent induces molecular homolysis and subsequent polymerization (Equations S1–S7), yielding a highly elastic polyether‐based polymer matrix. (2) Enrichment of inorganic components (Hard Layer): NaF and Na_2_O generated from the reaction of NaBF_4_ with trace impurities (Equations S13–S18) are uniformly dispersed within the polymer matrix [45]. This distribution mimics the hard inorganic layer‐reinforced soft organic layer architecture in seashells, effectively enhancing the structural stability of the SEI.

**FIGURE 4:**
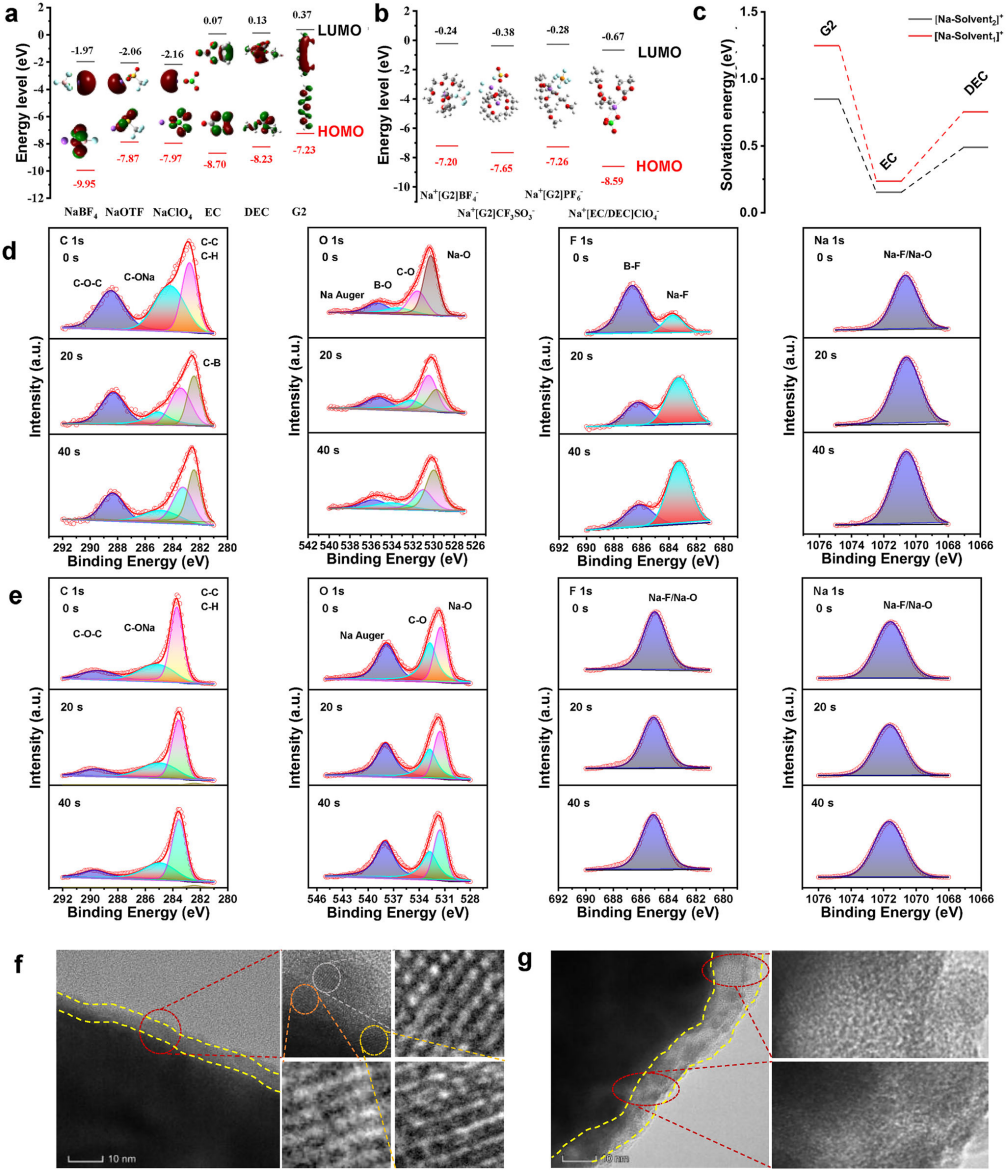
Theoretical analysis of interfacial chemistry and ex situ/in situ surface component characterizations of the biomimetic SEI. (a,b) Frontier molecular orbital energy levels (HOMO/LUMO) of common salts (NaBF_4_, NaOTF, NaClO_4_) and solvents (EC, DEC, G2); (c) Solvation energy barriers along different solvation and reduction pathways; (d,e) XPS depth‐profiling spectra (0, 20, and 40 s sputtering) showing the chemical component evolution of the SEI in the BG system vs. the control system, covering C 1s, O 1s, F 1s, and Na 1s core levels; (f,g) HR‐TEM images and enlarged lattice fringe details revealing the microscopic crystalline structure at the interfacial region.

XPS depth‐profiling analyses provide decisive experimental evidence for the longitudinal distribution of interfacial chemical components. In the BG system (Figure 4d), the C─O─C bonds characteristic of polyethers and the C─O─Na peaks of organic sodium salts in C 1s and O 1s spectra maintain highly consistent intensity and distribution across different sputtering depths, directly confirming that the flexible organic elastic layer achieves deep and uniform penetration within the SEI film [46]. Meanwhile, prominent Na─F binding energy peaks in the F 1s spectra mutually corroborate with Na─F/Na─O peaks in the Na 1s spectra, indicating that inorganic nanocrystals are uniformly embedded within the organic matrix. In stark contrast, the conventional electrolyte system (Figure 4e) exhibits XPS signals that decay rapidly with sputtering depth, coupled with feeble F elemental signals, failing to establish a robust inorganic–organic hybrid network. High‐resolution transmission electron microscopy (HR‐TEM) characterization further intuitively displays the hierarchical structure of the biomimetic SEI film (Figure 4f,g), featuring a dense surface and high crystallinity that completely match the theoretically predicted structure. To further examine the interfacial chemistry after prolonged cycling, XPS depth profiling was performed on µ‐Bi electrodes after 60 cycles (Figure S16). Spectra were collected before sputtering and after 20 and 40 s of sputtering. The fitted components in the C 1s, Na 1s, F 1s, and O 1s regions evolve with sputtering time, indicating chemical heterogeneity across the analyzed near‐surface and subsurface regions. Differences in the Cl 2p and B 1s signals between the control and BG systems further reflect differences in their interfacial chemical compositions. The depth‐dependent spectral evolution provides additional evidence that the interphases formed in the two electrolytes differ chemically after prolonged cycling. As the sputtering time was not converted into a calibrated absolute depth, these results are interpreted as comparative depth profiles rather than a quantitative determination of the full SEI thickness [47, 48].

To obtain additional spectroscopic information on the proposed interfacial chemistry, FTIR and Raman spectra were collected from µ‐Bi electrodes in the BG and NED electrolytes at different electrochemical states (Figure S17). The FTIR spectra exhibit potential‐ and cycling‐dependent changes in the fingerprint region associated with C─O─C/C─O vibrations, together with changes in the region associated with aliphatic C─H vibrations. These features are compatible with the presence and evolution of G2‐derived organic species at the electrode–electrolyte interface. The corresponding spectral evolution differs from that observed in the conventional NED system. The Raman spectra provide complementary information on the evolution of the electrode interface during electrochemical cycling. Because residual electrolyte species and the conductive carbon additive may also contribute to the measured signals, the individual spectral features are not considered standalone proof of a unique polymerization pathway. Instead, the spectroscopic observations are interpreted together with the DFT calculations, XPS depth profiles, and HR‐TEM characterization. Collectively, these results support the proposed formation of a polyether‐like organic component within the BG‐derived interphase. Cross‐validation between DFT calculations and XPS/HR‐TEM experiments is consistent with the BG electrolyte in situ constructing an elastic SEI with a biomimetic seashell structure on the micron‐sized bismuth surface via specific reduction decomposition pathways. This interface maintains film integrity through organic polymer components while ensuring rapid sodium ion transport via inorganic nanocrystal components.

### 2.5 Reaction Kinetics and Nanomechanical Mechanism

Morphological comparisons via SEM before and after cycling reveal macroscopic structural stability disparities. In the NED electrolyte, severe through‐cracking and pulverization emerged on the surface of micron‐sized bismuth particles after only 3 cycles (Figure 5a); whereas in the BG system, even after 60 extended cycles, the electrode surface preserved flat, dense, and exceptional integrity without any macroscopic cracks (Figure 5b), with electrode particles eventually evolving into grid‐like nanoparticle morphologies after prolonged cycling (Figure S18). This dramatic contrast in micro‐morphology directly reflects on electrochemical kinetic characteristics. Deconvolution analysis of electrochemical impedance spectroscopy (EIS) data based on distribution of relaxation times (DRT) techniques revealed that relaxation time peaks in the BG system shifted significantly toward the low‐frequency region, demonstrating marked reductions in charge transfer resistance and interfacial diffusion impedance (Figure 5c,d and Figure S19). Galvanostatic intermittent titration technique (GITT) measurements quantitatively evaluated ion diffusion rates (Figure 5e,f and Figure S20) across a wide voltage window from 0.2 to 0.8 V; the Na^+^ diffusion coefficient in the BG system consistently maintained a high magnitude of 10^−9^ cm^2^ s^−1^, whereas the control group experienced a severe order‐of‐magnitude plunge in diffusion coefficient during late‐stage cycling due to interfacial rupture. Combined with kinetic analyses from scan‐rate‐dependent CVs (Figure 5g and Figure S21), electrochemical impedance spectra (Figure S22), GITT chrono‐potentiometric response curves, and low activation energies derived from variable‐temperature impedance (Figure 5h and Figure S23), it is comprehensively confirmed that the biomimetic SEI film physically buffers volumetric stress while chemically constructing high‐speed sodium‐ion transport channels.

**FIGURE 5:**
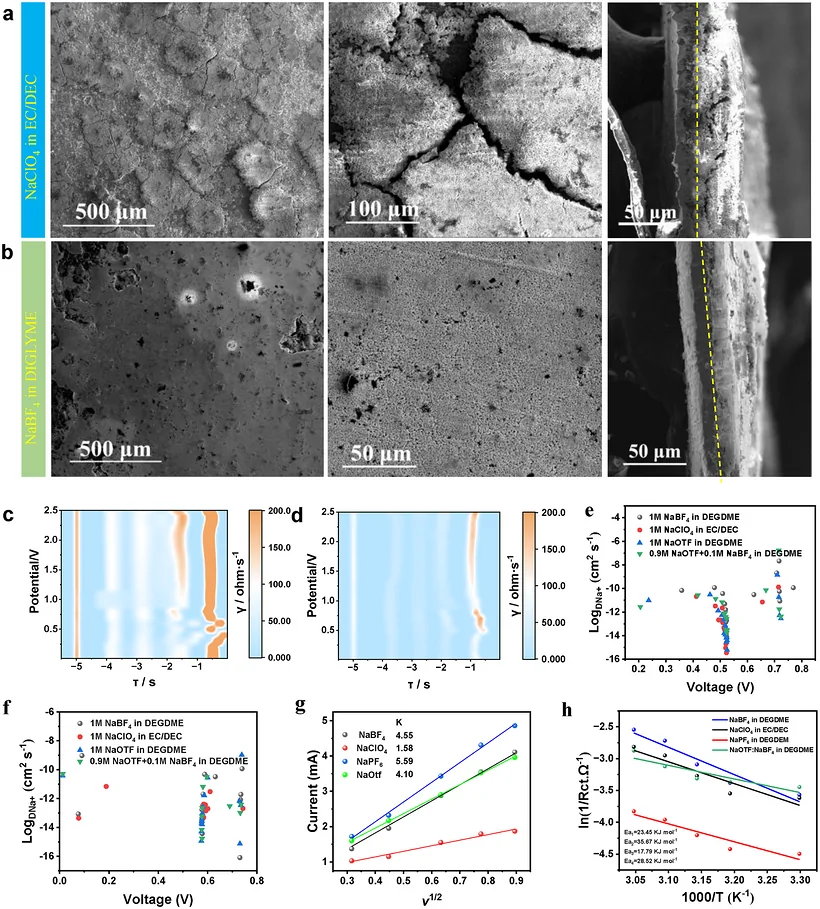
Morphological evolution and electrochemical kinetics analysis of the µ‐Bi electrodes. (a,b) SEM surface and cross‐sectional morphology images of electrodes cycled in the commercial electrolyte vs. the biomimetic electrolyte; (c,d) DRT analyses deconvoluting the impedance spectra to compare ion transport resistances; (e,f) Sodium‐ion diffusion coefficients derived from GITT measurements; (g) scan‐rate‐dependent CV analysis of reaction kinetics; (h) Arrhenius plots and apparent activation energies derived from variable‐temperature EIS.

To deeply dissect the physical essence of the biomimetic elastic interface, PeakForce QNM mode AFM was employed to map the nanomechanical properties of cycled electrode surfaces. Surface roughness statistics indicate that the SEI film surface in the BG system is exceptionally flat and uniform (R_a_ = 26 nm, R_q_ = 32 nm), far below the roughness of the control group (R_a_ = 54.7 nm, R_q_ = 70.8 nm, Figure 6a,b). The mechanical response of the interphase is closely related to its ability to accommodate the substantial volume changes of micron‐sized Bi during repeated sodiation and desodiation. The NED‐derived interphase exhibits a high DMT modulus of 1691 MPa, whereas the BG‐derived interphase exhibits a substantially lower value of 334 MPa (Figure 6c,d). Importantly, the lower modulus of the BG‐derived interphase is accompanied by a broad, smooth nonlinear deformation region in the force–displacement response, in contrast to the more abrupt fracture behavior observed for the NED‐derived interphase (Figure 6e,f). These results suggest that the BG‐derived interphase can accommodate local deformation more progressively rather than concentrating stress at the interface. During repeated alloying and dealloying, a brittle interphase may crack under the deformation of Bi particles, exposing fresh electrode surfaces to the electrolyte and promoting further interphase reconstruction. Such repeated damage can progressively compromise interfacial continuity and electrical contact. In contrast, the compliant organic component of the BG‐derived interphase is expected to accommodate part of the deformation, while the reinforcing inorganic domains contribute structural support. This combination can reduce localized stress concentration and help maintain interfacial integrity during cycling. The lower surface roughness and improved electrode morphology observed for the BG system are consistent with this interpretation. Thus, the mechanical advantage of the biomimetic interphase arises from the balance between deformation accommodation and structural reinforcement, rather than from maximizing the stiffness of the SEI alone [49, 50]. To determine whether the interfacial mechanical differences persist after prolonged cycling, additional AFM characterization was performed on µ‐Bi electrodes after 60 cycles (Figure S24). The DMT modulus of the BG‐derived interphase is 354 MPa, lower than the 487 MPa measured for the control interphase. The corresponding adhesion forces are 4.28 and 10.8 nN, respectively. The AFM height profiles also show a smaller vertical variation for the BG‐derived surface than for the control surface. These results indicate that the two interphases retain distinct morphological and mechanical characteristics after repeated electrochemical cycling. The lower measured modulus of the BG‐derived interphase is consistent with a more compliant mechanical response, which may help accommodate local deformation during repeated alloying and dealloying. Together with the XPS depth‐profiling results, these observations provide complementary evidence for the distinct chemical and mechanical characteristics of the BG‐derived interphase after prolonged cycling.

**FIGURE 6:**
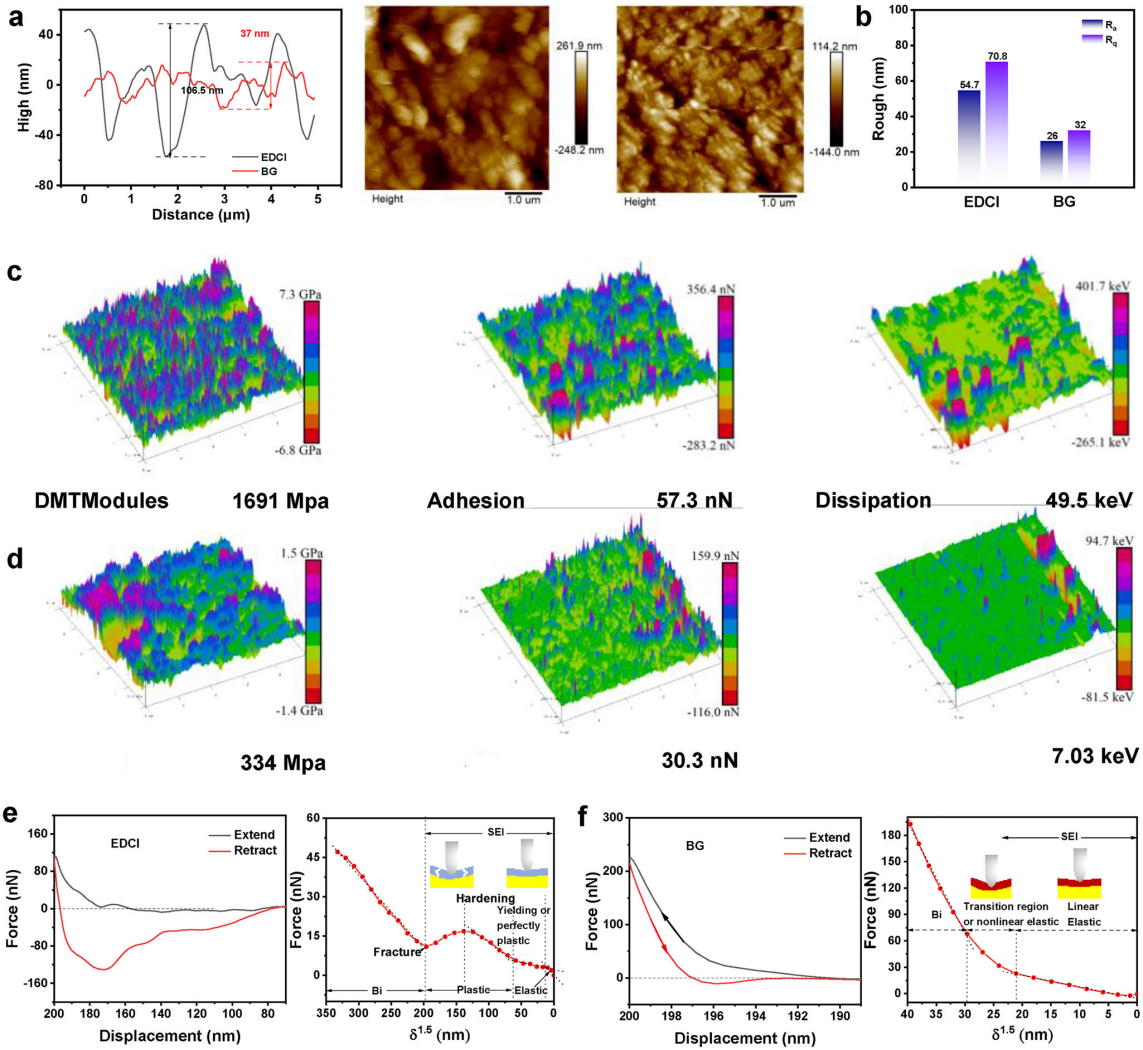
Nanomechanical property characterizations of the biomimetic SEI membrane. (a) AFM morphology maps of SEI surfaces for the commercial control group and the BG; (b) Corresponding spatial distribution maps of DMT Modulus, Adhesion force, and Energy Dissipation; (c) Quantitative column charts comparing surface roughness (R_a_ and R_q_); (d) Force‐Displacement curves and contact mechanics models derived from nanoindentation experiments, illustrating the transition and elastic behavior under mechanical probing.

To further evaluate the feasibility of the designed electrolyte beyond half‐cell measurements, a full cell was assembled using a µ‐Bi anode paired with Na_3_V_2_(PO_4_)_3_ (NVP). The full‐cell performance was evaluated at 25°C. As shown in Figure S25a,b, the cell was cycled at 1 C for 60 cycles, and the corresponding charge–discharge profiles were recorded at selected cycle numbers. The Coulombic efficiency approaches approximately 100% after the initial cycles, while the discharge capacity gradually decreases during cycling. The rate capability was further evaluated at current rates of 0.1, 0.2, 0.3, 1, 2, and 3 C (Figure S25c,d). The discharge capacity decreases as the current rate increases, providing an initial assessment of the rate‐dependent electrochemical behavior of the full cell. These results extend the evaluation of the BG electrolyte from half‐cell measurements to a full‐cell configuration. Nevertheless, the capacity decay observed during cycling indicates that further optimization of electrode balancing, cathode compatibility, and electrolyte quantity is required before practical performance can be fully established.

## 3 Conclusion

This study successfully resolves the volume expansion and interfacial failure issues of µ‐Bi anodes during wide‐temperature cycling through microscopic solvation structure regulation and biomimetic interfacial engineering. Inspired by the microstructures of natural seashells, a BG ether‐based electrolyte system was designed, successfully constructing an in situ biomimetic elastic SEI film possessing both a soft organic buffer layer and a hard inorganic scaffold on the µ‐Bi surface. Theoretical calculations and XPS/HR‐TEM characterizations confirm that the free‐radical polymerization products of the G2 solvent and NaF nanocrystals generated from NaBF_4_ reduction synergistically constitute a robust and dense hybrid network, endowing the SEI film with a low Young's modulus of only 334 MPa and a broad non‐linear elastic transition region capable of efficiently dissipating mechanical stress. This biomimetic interface equips the µ‐Bi anode with exceptional kinetic characteristics and long‐term stability, achieving 100% capacity retention after 450 cycles at 5 C within the voltage window of 0.01–1.5 V. Furthermore, stable cycling for 2000 cycles at 5 C is demonstrated within a restricted voltage window of 0.01–1.0 V, outstanding performance under a high mass loading, and excellent wide‐temperature cycling and rate responses under extreme low temperatures of −40°C. More broadly, this work suggests a design principle for electrolyte‐derived interphases on alloy anodes: solvent and salt components should be selected not only according to their solvation and transport properties, but also according to their complementary interfacial decomposition pathways and the mechanical functions of the resulting products. Rather than maximizing SEI stiffness, an effective interphase should balance a compliant organic matrix that accommodates strain with reinforcing inorganic domains that help preserve structural integrity and Na^+^ transport. This principle provides a potential direction for developing mechanically adaptive interphases for other alloy anodes undergoing large volume changes, although its applicability to different electrode and electrolyte systems requires further validation.

## Author Contributions

**Weijia Meng** and **Zhenzhen Dang** conceived the idea, designed the project, and wrote the manuscript. **Jing Li** and **Jiayue Gao** prepared the electrolyte, performed electrochemical measurements, and analyzed the data. **Qiuyu Sun** and **Ning Pan** contributed to DFT and MD calculations and formal data analysis. **Yangyang Li** and **Hongqing Yin** provided partial material characterization and testing. **Jingfeng Ning** supervised the research, discussed the mechanistic results, and commented on the manuscript. **Jiaye Ye** contributed to writing – review and editing.

## Conflicts of Interest

The authors declare no conflicts of interest.

## Supporting information




**Supporting File**: advs78740‐sup‐0001‐SuppMat.docx.

## Data Availability

The data that support the findings of this study are available from the corresponding author upon reasonable request.
