# Supplementary material for: A Biomimetic Elastic SEI Film Enables Micron‐Sized Bismuth Anodes for Wide‐Temperature Sodium‐Ion Batteries
Source: Adv Sci (Weinh). 2026 Nov 9:e78740. Online ahead of print. doi: 10.1002/advs.78740 (PMC13748907; doi:10.1002/advs.78740)
Supplement: Supplementary file 1 [file ADVS-9999-e78740-s001.docx]

**Supporting Information**

**A Biomimetic Elastic SEI Film Enables Micron-Sized Bismuth Anodes for Wide-Temperature Sodium-Ion Batteries**

**Zhenzhen Dang^a^, Jing Li^b^, Jiayue Gao^b^, Qiuyu Sun^b^, Ning Pan^b^, Yangyang Li^b^, Jingfeng Ning^a^, Hongqing Yin^c^, Jiaye Ye^d,^ *, Weijia Meng^b,^ ***

^a^ School of Smart City and New Energy, Xi'an Jiaotong University City College, Xi'an, 710064, China

^b^ Shaanxi Provincial Key Laboratory of New Transportation Energy and Automotive Energy Saving, School of Energy and Electrical Engineering, Chang'an University, Xi'an, 710064, China

^c^ China Energy Engineering Group Hydrogen Energy Co., Ltd., Beijing, 100000, China

^d^ School of Chemistry and Physics, Faculty of Science, Queensland University of Technology, 2 George Street, QLD 4000, Australia

**Experimental Section**

**1. Materials and Reagents**

Commercial micron-sized bismuth powder (μ-Bi) was used as the active material. The pristine morphology and phase composition of the commercial μ-Bi powder were characterized by SEM and XRD. Super P carbon black was used as the conductive additive, poly(vinylidene fluoride) (PVDF) as the binder, and N-methyl-2-pyrrolidone (NMP) as the dispersing solvent. Copper foil was used as the current collector, glass fiber membrane as the separator, and sodium metal foil as both the counter and reference electrode. CR2032 coin-type cells were used for electrochemical assembly.

The electrolyte solvents included diethylene glycol dimethyl ether (DEGDME, G2), ethylene carbonate (EC), and diethyl carbonate (DEC). The sodium salts included NaBF_4_, NaClO_4_, NaPF_6_, and NaOTF. All salts were vacuum-dried before use, and the solvents were dried over molecular sieves. Electrolyte preparation and cell assembly were carried out in an Ar-filled glovebox with both H_2_O and O_2_ contents below 0.1 ppm.

**2. Electrolyte Preparation**

The optimized biomimetic electrolyte, denoted as BG, was prepared by dissolving NaBF_4_ in DEGDME to obtain 1 M NaBF_4_/DEGDME. The conventional commercial electrolyte, denoted as NED, was prepared by dissolving NaClO_4_ in an EC/DEC mixed solvent to obtain 1 M NaClO_4_/EC/DEC. For comparison, additional electrolytes were also prepared, including 1 M NaPF_6_/DEGDME, 1 M NaOTF/DEGDME, and a mixed-salt electrolyte of 0.9 M NaOTF + 0.1 M NaBF_4_ in DEGDME.

In a typical preparation, the desired amount of sodium salt was weighed and added to the corresponding solvent, followed by sealing and stirring for 12 h until a clear and homogeneous solution was obtained. The resulting electrolytes were stored in the glovebox before use to avoid moisture and air exposure.

**3. Electrode Fabrication and Cell Assembly**

The μ-Bi working electrodes were fabricated by mixing μ-Bi active material, Super P carbon black, and poly(vinylidene fluoride) (PVDF) binder at a mass ratio of 8:1:1 in N-methyl-2-pyrrolidone (NMP) to form a homogeneous slurry. The slurry was uniformly coated onto Cu foil and dried under vacuum at 60-80 °C for 12 h. For the high-mass-loading tests, the μ-Bi active-material loading was 9.49 mg cm^-2^, corresponding to an areal capacity of 3.2 mAh cm^-2^. CR2032 half-cells were assembled using the μ-Bi electrode as the working electrode, sodium metal foil as the counter/reference electrode, and a glass fiber membrane as the separator. The electrolyte volume was 80 μL per half-cell. The assembled cells were rested for 6-12 h before electrochemical testing to ensure sufficient electrolyte wetting. For the Na||Bi half-cell configuration, an N/P ratio is not applicable because sodium metal was used as the counter/reference electrode. For full-cell measurements, the μ-Bi anode was paired with a NVP cathode. The cathode active-material loading was 3 mg cm^-2^, and the μ-Bi anode active-material loading was 1 mg cm^-2^. The electrolyte volume was 80 μL per full cell. The negative-to-positive capacity ratio (N/P ratio) was 1.2, calculated from the reversible areal capacities of the μ-Bi anode and the cathode, and the 1 C corresponding to 385 mA g^-1^. The full-cell specific capacity was normalized to the mass of μ-Bi anode.

**4. Electrochemical Measurements**

Cyclic voltammetry (CV) was performed over a voltage range of 0.01–1.5 V vs. Na/Na^+^ at scan rates of 0.1, 0.2, 0.4, 0.6, and 0.8 mV s^-1^ to evaluate the alloying/dealloying reversibility of μ-Bi and the surface-controlled pseudocapacitive contribution. Galvanostatic charge–discharge (GCD) tests were carried out over 0.01–1.5 V vs. Na/Na^+^, where 1 C = 385 mA g^-1^. At 25 °C, rate performance was evaluated at stepwise current densities including 0.1 C, 0.125 C, 0.3 C, 0.5 C, 1 C, 3 C, 5 C, 8 C, and 10 C. Long-term cycling was conducted at 0.1 C and at a high rate of 5 C. The μ-Bi electrode delivered a capacity retention of 100% after 450 cycles at 5 C at 25 °C. In a restricted voltage window of 0.01–1 V, the electrode maintained a discharge capacity of 380 mAh g^-1^ at 0.125 C and completed 2000 ultra-long cycles at 5 C. High-mass-loading tests were performed at 25 °C with an active material loading of 9.49 mg cm^-2^ to evaluate cycling stability under high areal capacity. Low-temperature and wide-temperature tests were conducted at −40 °C at 0.1 C, and variable-temperature rate tests were performed from −25 °C to −40 °C. The cell maintained stable charge–discharge plateaus and cycling stability under these extreme conditions. Electrochemical impedance spectroscopy (EIS) was measured over a frequency range of 100 kHz to 0.01 Hz with an AC amplitude of 5 mV. The impedance data were used for Arrhenius fitting, ionic conductivity calculation, and distribution of relaxation times (DRT) analysis. DRT analysis was employed to deconvolute the charge-transfer resistance and interfacial diffusion impedance. Galvanostatic intermittent titration technique (GITT) was performed using constant-current pulses followed by relaxation periods. The Na^+^ diffusion coefficient was calculated from the GITT voltage–time curves. In the BG system, the Na^+^ diffusion coefficient remained on the order of 10⁻⁹ cm² s^-1^ over a wide voltage range of 0.2–0.8 V. Linear sweep voltammetry (LSV) was conducted at −40 °C to evaluate the electrochemical stability window of the electrolyte. The BG system exhibited a wide stability window of >4.2 V vs. Na/Na^+^ without obvious electrolyte decomposition. Symmetric cell tests were performed using Na||Na cells with the BG electrolyte and the commercial control electrolyte. The cells were cycled galvanostatically for up to 700 h to compare polarization stability, dendrite suppression, and interfacial robustness. Ionic conductivity measurements were carried out by variable-temperature EIS from 45 °C to −45 °C. The bulk resistance of the electrolyte was extracted and used to calculate ionic conductivity, followed by Arrhenius fitting to obtain the activation energy for ion transport.

**5. Materials Characterization**

X-ray diffraction (XRD) was performed with Cu Kα radiation over a 2θ range of 10-80°. The pristine μ-Bi powder and cycled electrodes were analyzed. For ex-situ XRD, electrodes were retrieved at selected charge/discharge potential nodes in an Ar-filled glovebox and sealed before measurement to track the reversible phase evolution of Bi, intermediate Na-Bi phases such as Na_3_Bi, and the recovery of the initial Bi phase. Scanning electron microscopy (SEM) was used to observe the surface and cross-sectional morphologies of the pristine μ-Bi powder and the electrodes before and after cycling. Cross-sectional samples were prepared and imaged to evaluate electrode structural integrity and SEI cracking behavior. High-resolution transmission electron microscopy (HR-TEM) was employed to characterize the microstructure of the cycled SEI film. The SEI was scraped from the cycled electrode in an Ar-filled glovebox, dispersed in anhydrous ethanol, dropped onto a Cu grid, vacuum-dried, and transferred under inert atmosphere. Lattice fringes and crystalline domains corresponding to inorganic nanophases embedded in the organic matrix were analyzed. X-ray photoelectron spectroscopy (XPS) was performed with an Al Kα source. Binding energies were calibrated using the C 1s peak at 284.8 eV. Depth profiling was conducted with Ar^+^ sputtering for 0 s, 20 s, and 40 s. High-resolution C 1s, O 1s, F 1s, and Na 1s spectra were collected to analyze the longitudinal distribution of polyether-based organic sodium salts, NaF, Na_2_O, and other SEI components. Atomic force microscopy (AFM) was carried out in PeakForce QNM mode to map the nanomechanical properties of the cycled electrode surfaces, including DMT modulus, adhesion force, energy dissipation, and surface roughness. Force–displacement curves were analyzed to evaluate elastic deformation, nonlinear transition behavior, and brittle fracture. Differential scanning calorimetry (DSC) was performed on sealed electrolyte samples under N_2_ atmosphere from -100 °C to 40 °C at a heating rate of 10 °C min^-1^. The FTIR and Raman spectroscopy were employed to investigate the potential-dependent evolution of interfacial species on μ-Bi electrodes. Electrodes in the BG and NED electrolytes were collected at the initial state, at 0.01 V, at 1.5 V, and after three cycles. After electrochemical testing, the electrodes were retrieved in an Ar-filled glovebox and transferred for spectroscopic analysis without rinsing.

**6. Theoretical Calculations**

Density functional theory (DFT) calculations were performed to evaluate the frontier molecular orbital energy levels (HOMO/LUMO), spatial charge distribution, bond dissociation energies (BDEs), solvation energy barriers, and reduction decomposition pathways of the solvent and salt molecules in different electrolyte systems. Calculations were carried out using Gaussian 16 at the B3LYP/6-311+G(d, p) level, with an implicit solvation model. The BDE was calculated as:

BDE= *E*(A)+*E*(B)−*E*(A−B)

Where *E*(A−B) is the energy of the parent molecule and *E*(A) and *E*(B) are the energies of the corresponding radical fragments. The BDE analysis revealed that C1–O1 and C2–O1 bonds in the G2 molecule have low homolytic cleavage energies of 3.72 eV and 3.76 eV, respectively, which can trigger in-situ radical polymerization at low potentials. In contrast, EC in the commercial NED electrolyte mainly undergoes ring-opening decomposition to form rigid alkyl sodium carbonate dimers and Na_2_CO_3_ particles.

Solvation energy and reduction pathway calculations were conducted to analyze the coordination structure of Na^+^, the participation of BF_4_^-^ anions, and the reduction decomposition of G2 and NaBF_4_. Combined with the reaction pathways in the Supporting Information (Equations S1–S20), the formation mechanism of the biomimetic SEI was deduced: G2 molecules undergo homolysis and radical coupling to generate a flexible polyether-based organic sodium salt matrix, while NaBF_4_ reduction and reactions with trace impurities produce NaF and Na_2_O inorganic nanocrystals that serve as the hard inorganic scaffold.

Ab initio molecular dynamics (AIMD) simulations were performed for 1 M NaBF_4_/DEGDME and 1 M NaClO_4_/EC+DEC systems. The simulations used the PBE exchange–correlation functional, PAW pseudopotentials, a plane-wave cutoff of 500 eV, the NVT ensemble, a Nosé–Hoover thermostat at 300 K, a time step of 1 fs, and a total simulation time of 10 ps. The fluctuations of total energy and temperature versus time confirmed that the simulated systems reached thermodynamic equilibrium. The radial distribution functions *g*(*r*) and coordination numbers of Na^+^ with solvent oxygen atoms and anions were calculated from the AIMD trajectories to reveal the Na^+^ solvation structure and first solvation shell composition in the BG and NED systems.

**7. Proposed reactions pathways of electrolyte decompositions**

Decomposition of NaBF_4_ or NaPF_6_ (M = B or P),

$NaMF_{n}\rightleftharpoons NaF+MF_{n-1}$	(S1)

**Diglyme,**

$+e^{-}+Na^{+}\underset{\to}{BF_{3}}CH_{3}Na+$	(S2)

which refers to the homolysis regarding C1-O1 in Fig. S4.

$+e^{-}+Na^{+}\underset{\to}{BF_{3}}CH_{3}ONa+$	(S3)

which refers to the homolysis regarding C2-O1 in Fig. S4.

$+e^{-}+Na^{+}\underset{\to}{BF_{3}}CH_{3}Na+$	(S4)

which refers to the homolysis regarding C1-O1 in Fig. S4.

$$+n\left( \right)e^{-}+Na^{+}\underset{\to}{BF_{3}}CH_{3}Na$$

$+$	(S5)

$+2e^{-}+2Na^{+}\underset{\to}{BF_{3}}2CH_{3}ONa+$	(S6)

which refers to the homolysis regarding C2-O1 in Fig. S4.

$$2n+2e^{-}+2Na^{+}\underset{\to}{BF_{3}}+$$

$CH_{2}=CH_{2}\uparrow$	(S7)

**EC,**

$2+2e^{-}+2Na^{+}\to(CH_{3}OCO_{2}Na)_{2}+CH_{2}=CH_{2}\uparrow$	(S8)

$+2e^{-}+2Na^{+}\to(NaCH_{2}CH_{2}OCO_{2}Na)_{2}+CH_{2}=CH_{2}\uparrow$	(S9)

 + 2e^−^ + 2Na^+^ → Na_2_CO_3_ + 2CH_2_=CH_2_↑	(S10)

**H_2_O,**

H_2_O + e^−^ + Na^+^ → NaOH + ½H_2_↑	(S11)

NaOH + e^−^ + Na^+^ → Na_2_O + ½H_2_↑	(S12)

**CO_2,_**

2CO_2_ + 2e^−^ + 2Na^+^ → Na_2_CO_3_ + CO↑	(S13)

**H_2_O and EC,**

H_2_O + (CH_2_OCO_2_Na)_2_ → Na_2_CO_3_ + CO_2_↑ + (CH_2_OH)_2_	(S14)

**H_2_O and NaBF_4_ or NaPF_6_ (M = B or P)**

MF_n−1_ + H_2_O → 2HF + MF_n−3_O	(S15)

MF_n−3_O + ke^−^ + kNa^+^ → NaF + Na_x_MOF_y_	(S16)

Note that Eq. S18 does not show the exact stoichiometry, but rather the basic equations.

**H_2_O, NaBF_4_ and EC,**

2HF + (CH_2_OCO_2_Na)_2_ → 2NaF + (CH_2_OCO_2_H)_2_	(S17)

**H_2_O, NaBF_4_, and EC/CO_2,_**

2HF + Na_2_CO_3_ → 2NaF + H_2_CO_3_	(S18)

**H_2_O, NaBF_4_ and DMC,**

CH_3_OCO_2_Na + HF → NaF + CH_3_OCO_2_H	(S19)

CH_3_ONa + HF → NaF + CH_3_OH	(S20)

**Supporting Information Figure Captions (Figures S1 – S25)**

**Figure S1.** Calculated bond dissociation energies (BDE) and cleavage pathways for the G2 (DEGDME) solvent molecule.

**Figure S2.** Calculated bond dissociation energies and ring-opening decomposition pathways for the EC solvent molecule.

**Figure S3.** Optimized molecular structures and geometric configurations of various electrolyte components and reduction intermediates.

**Figure S4.** Energy and temperature fluctuations over time during the AIMD calculations for: (a) 1 M NaBF_4_ in DEGDME, and (b) 1 M NaClO_4_ in EC/DEC.

**Figure S5.** SEM images at different magnifications and the corresponding XRD pattern of pristine commercial bismuth powder (μ-Bi).

**Figure S6.** Galvanostatic charge-discharge voltage profiles of the μ-Bi electrode at 0.1 C in the commercial 1 M NaClO_4_ in EC/DEC electrolyte.

**Figure S7.** Detailed charge-discharge voltage profiles corresponding to the rate performance test of the μ-Bi electrode.

**Figure S8.** Long-term high-capacity cycling performance of the μ-Bi electrode at 25℃ with a high mass loading.

**Figure S9.** Long-term galvanostatic cycling curves of symmetrical cells comparing the BG electrolyte and the commercial control electrolyte.

**Figure S10.** Electrochemical performance of commercial μ-Bi electrodes tested within a restricted voltage window of 0.01-1 V in 1 M NaBF_4_ / DEGDME: (a) charge-discharge curves, (b) cycling performance at 0.125 C, (c) voltage profiles at various rates, (d) rate capability, and (e) long-term cycling stability at 5 C.

**Figure S11.** Electrochemical performance of μ-Bi electrodes in alternative single-salt electrolytes: (a, b) 1 M NaPF_6_ in DEGDME, and (c, d) 1 M NaOTF in DEGDME.

**Figure S12.** Electrochemical performance of μ-Bi electrodes in the mixed-salt electrolyte (0.9 M NaOTF + 0.1 M NaBF_4_ in DEGDME): (a) charge-discharge profiles, (b) cycling at 0.1 C, (c) cycling at 1 C, (d) cycling at 5 C, (e) voltage profiles at various rates, and (f) rate capability.

**Figure S13.** Low-temperature electrochemical characteristics and corresponding dQ/dV differential capacity curves of the μ-Bi electrode.

**Figure S14.** Initial charge–discharge capacities and initial Coulombic efficiencies of μ-Bi electrodes in different electrolytes: 1 M NaBF_4_/DEGDME, 1 M NaPF_6_/DEGDME, 1 M NaOTf/DEGDME, and 0.9 M NaOTf + 0.1 M NaBF_4_/DEGDME.

**Figure S15.** XRD patterns of the μ-Bi electrode collected at various selected states of charge/discharge (at specific potential cutoffs) to investigate the reversible phase evolution.

**Figure S16.** XPS depth profiles of μ-Bi electrodes after 60 cycles in the control and BG electrolytes. (a) Control electrolyte and (b) BG electrolyte. High-resolution C 1s, Na 1s, F 1s, O 1s, and electrolyte-related Cl 2p or B 1s spectra were collected before sputtering and after 20 and 40 s of sputtering to investigate the depth-dependent chemical composition of the interphases.

**Figure S17.** FTIR and Raman spectra of μ-Bi electrodes in the NED and BG electrolytes at the initial state, at 0.01 V, at 1.5 V, and after three cycles. (a, b) FTIR spectra of electrodes in the NED and BG electrolytes, respectively. (c, d) Raman spectra of BG-derived electrodes in the 0-500 and 1200-1700 cm^-1^ regions, respectively. (e, f) Raman spectra of NED-derived electrodes in the corresponding regions.

**Figure S18.** Ex-situ SEM morphology images of μ-Bi electrodes after cycling under different conditions (initial state and after 3rdcycle).

**Figure S19.** DRT impedance deconvolution analysis curves measured at different potentials.

**Figure S20.** GITT potential response curves and calculated ionic diffusion coefficients for various electrolyte systems.

**Figure S21.** CV curves measured at various scan rates for determining surface-controlled pseudocapacitive contributions and ion diffusion kinetics.

**Figure S22.** Variable-temperature electrochemical impedance spectra and Arrhenius linear fits used for calculating the apparent activation energies (E_a_) of different electrolyte systems.

**Figure S23.** Electrochemical impedance spectra (Nyquist plots with high-frequency enlarged views) comparing the NaClO_4_ / EC/DEC and NaBF_4_ / DEGDME systems at room temperature.

**Figure S24.** AFM characterization of μ-Bi electrodes after 60 cycles in the control and BG electrolytes. (a) Representative height profiles and surface topography maps; (b) comparison of surface roughness parameters; (c, d) DMT modulus, adhesion force, and energy-dissipation maps of the control and BG-derived interphases, respectively; (e, f) corresponding force–displacement curves for the control and BG-derived interphases, respectively.

**Figure S25.** Electrochemical performance of the full cell assembled using a μ-Bi anode and NVP cathode. (a) Cycling performance at 1 C and 25 °C. (b) Charge–discharge profiles at selected cycle numbers. (c) Rate capability at 0.1, 0.2, 0.3, 1, 2, and 3 C. (d) Corresponding charge-discharge profiles at different current rates.
